# Postoperative Lung Function Advantages in Pulmonary Segmentectomy for Early‐Stage Lung Cancer

**DOI:** 10.1111/1759-7714.15515

**Published:** 2025-01-26

**Authors:** Ernesto Crisafulli, Claudio Micheletto, Alessio Campisi, Emanuele Vocale, Chiara Schiena, Giulia Sartori, Gianluca Gaburro, Elide Felici, Maurizio Infante

**Affiliations:** ^1^ Department of Medicine, Respiratory Medicine Unit University of Verona and Azienda Ospedaliera Universitaria Integrata of Verona Verona Italy; ^2^ Pulmonology Unit Verona Integrated University Hospital Verona Italy; ^3^ Department of Thoracic Surgery University and Hospital Trust‐Ospedale Borgo Trento Verona Italy

**Keywords:** diffusion capacity for carbon monoxide, early‐stage lung cancer, long‐term evaluation, lung function, segmentectomy

## Abstract

In early‐stage lung cancer, lung function appears to be less compromised after segmentectomy than lobectomy, though the advantage seems modest. We aimed to re‐assess postoperative lung function in surgical patients, with a particular focus on the diffusion capacity for carbon monoxide (DL_CO_). We evaluated all patients who underwent either lobectomy or segmentectomy for T1a‐c lung cancer at our center between March 2016 and March 2023. From January to June 2024, patients who had undergone segmentectomy, along with a matched cohort of patients who had undergone lobectomy, were invited for a repeat lung function evaluation. Patients were matched 1:1 based on age, sex, surgical approach, year in which the procedure was performed, and tumor location. Lung function testing data including DL_CO_ were then compared to preoperative measures. During the study period, 480 patients received a lobectomy, and 97 received a segmentectomy. Complete lung function evaluation for the study was available for 52 patients (26 matched pairs). The median time from lung resection to repeat spirometry was 35 months. A modest reduction of lung function measures was observed in the segmentectomy group. Conversely, all lung function measures, including DL_CO_, were significantly impaired in the lobectomy group. In early‐stage lung cancer, patients who perform segmentectomy demonstrated better long‐term lung function preservation compared to those who underwent lobectomy. Whenever feasible, segmentectomy should be considered the procedure of choice for early‐stage lung cancer patients.

## Graphical abstract

In patients with T1a‐c lung cancer, we repeat after 35 months post‐lung resection the lung function evaluation (including DL_CO_) of 26 patients undergoing segmentectomy matched with 26 patients undergoing lobectomy. Comparing data with preoperative measures, patients with segmentectomy resulted in better DL_CO_ preservation than those with lobectomy. We demonstrated that when feasible, segmentectomy should be considered the procedure of choice for patients with early‐stage lung cancer.

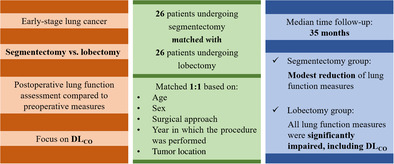

## 1 Introduction

Lung function, expressed mainly as forced expiratory volume in the first second (FEV_1_), appears to be less compromised after segmentectomy than lobectomy, but this advantage seems modest [1]. Consequently, it may be argued that the additional time and effort required to perform a segmentectomy, a technically more complex procedure, may not be justified. However, the retrospective nature and the high heterogeneity of published studies may limit the validity of such conclusions [1].

Parenchyma‐sparing lung resections, such as segmentectomy, have gained traction following two recent trials, the JCOG0802/WJOG4607L [2] and CALGB 140503 [3], supporting their oncological equivalence to lobectomy for early‐stage peripheral lung cancer [3].

At our Thoracic Surgery Unit of the University and Hospital Trust of Verona (Italy), segmentectomy has been offered to selected patients with T1a‐c lung cancer or with a single pulmonary lesion in patients with a history of extra‐pulmonary cancer or as a compromise procedure. Our impression was that patients undergoing segmentectomy had clinical and perceived symptom benefits in the postoperative period compared to lobectomy patients. In this study, we aimed to re‐assess postoperative lung function in post‐surgical patients, evaluating not only FEV_1_ and respiratory flows but also lung diffusion capacity.

## 2 Methods

All patients who had received a segmentectomy for early‐stage lung cancer in our Center from March 2016 to March 2023 and who did not have cancer recurrence were invited to receive repeat spirometry between January and June 2024. Patients who had received a segmentectomy were matched in a ratio of 1:1 to patients who had received a lobectomy based on age, sex, surgical approach (video‐assisted thoracoscopic surgery‐VATS or open surgery), year in which the surgical procedure was performed, and surgical location (same lobe). Both groups (segmentectomy and lobectomy) were invited to undergo a repeat lung function evaluation (defined as T_1_ assessment). For each segmentectomy patient, one lobectomy patient with matching characteristics was selected. Spirometry data were then compared to pre‐surgical assessment data (T_0_ assessment). Only patients with both T_0_ and T_1_ data available were included. Patients who had received a wedge resection and those who had received multimodality treatment were excluded.

Postoperative lung function was assessed at the Respiratory Medicine Unit of our Hospital following international recommendations [4]. A flow‐sensing spirometer connected to a computer for data analysis (Jaeger MasterScreen PFT System) was used. Forced vital capacity (FVC), FEV_1_ and total lung capacity (TLC) were recorded. The single‐breath method was used to measure the diffusion capacity for carbon monoxide (DL_CO_). FEV_1_, FVC, TLC, and DL_CO_ were expressed as absolute values and percentages of the predicted values.

The study protocol was approved by the local Ethics Committee (no. 3128CESC) and was conducted in accordance with Good Clinical Practice recommendations and the Declaration of Helsinki. Written informed consent was obtained from all patients.

A preliminary Shapiro–Wilk test was conducted to assess data distribution. Categorical variables are reported as percentages, while continuous variables are expressed as mean (SD) for normally distributed data or median [First quartile; Third quartile] for non‐normal distributed data. Categorical variables were compared using the Chi‐square test or the Fisher exact test. Continuous baseline variables between the two groups were compared by the independent *t*‐test or the non‐parametric Mann–Whitney, while the paired *t*‐test or the non‐parametric Wilcoxon signed‐rank test were used to compare the differences between T_0_ and T_1_. Lung function changes by segment location were compared using one‐way ANOVA with Bonferroni post hoc analysis.

All statistical analyses were performed using IBM SPSS, version 17.0 (IBM Corp., Armonk, NY, USA), with a significance threshold set at *p* < 0.05.

## 3 Results

**FIGURE 1:**
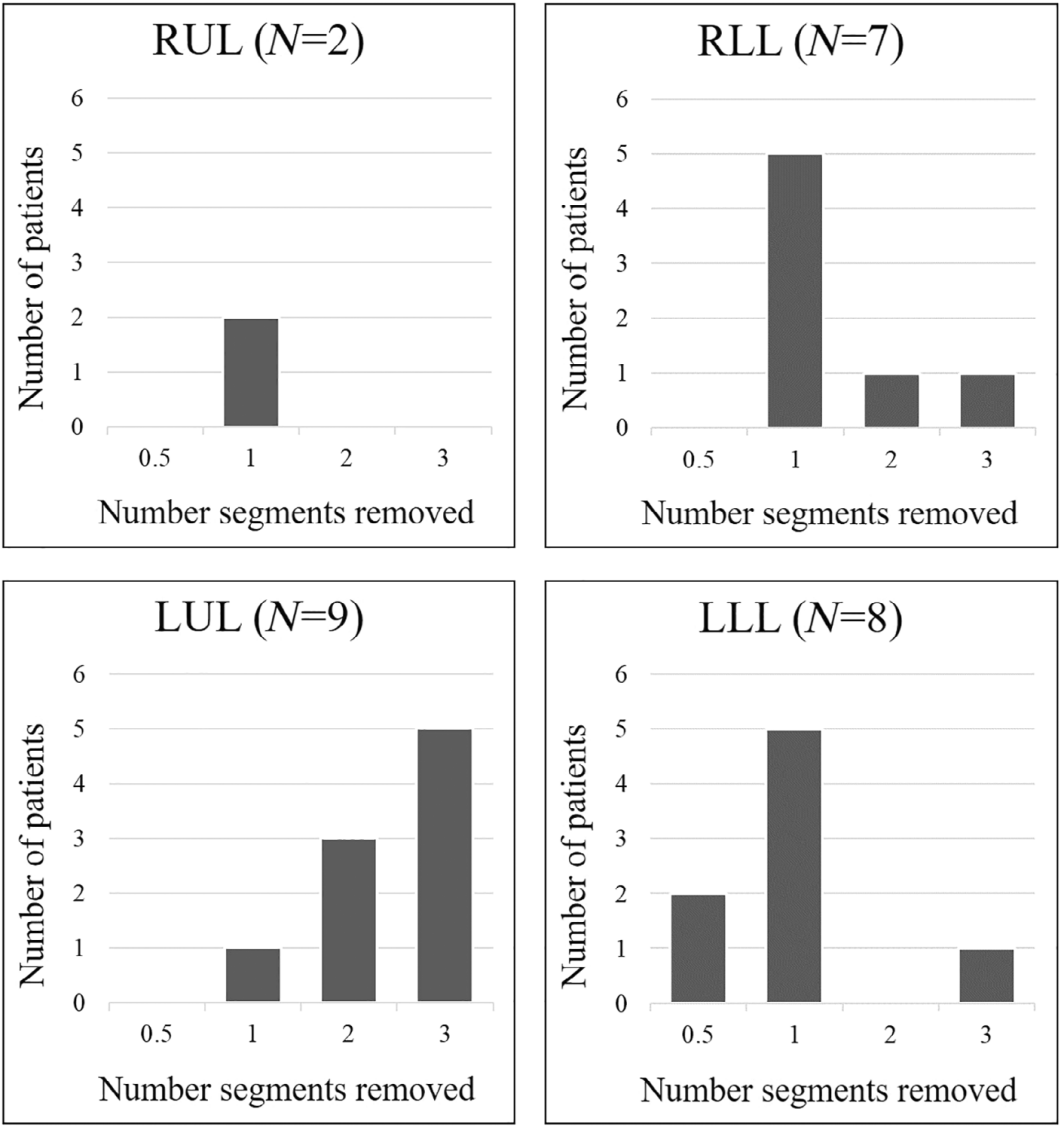
Frequency of segments removed for each lobe in patients undergoing segmentectomy. LLL, left lower lobe; LUL, left upper lobe; RLL, right lower lobe; RUL, right upper lobe.

During the study period, 480 patients received a lobectomy, and 97 received a segmentectomy. Eleven patients had 2–4 segments removed and 88 (91%) segmentectomies were carried out by VATS. As of January 2024, among 97 patients who had received a segmentectomy 15 (15%) had died, 6 (6.2%) were in poor clinical condition and unable to repeat spirometric testing, 33 (34%) declined spirometry at T_1_ and for 17 patients (17%) spirometric data at T_0_ were unavailable. The remaining 26 patients (27%) were matched with 26 similar patients undergoing lobectomy. Complete lung function evaluation for the study was available for 52 patients (26 matched pairs) (Table 1). In patients undergoing segmentectomy, the number of removed segments was higher in the left lung (*N* = 17) than in the right lung (*N* = 9); Figure 1 reports the number of removed segments by lobe and Table 2 provides information about the frequency of the segmentectomies procedures.

**TABLE 1:** General and lung function characteristics of patients according to the pulmonary surgery.

Variables	Patients undergoing segmentectomy (*N* = 26)			Patients undergoing lobectomy (*N* = 26)			*p*‐value at T_0_ between patients undergoing segmentectomy and lobectomy
	T_0_	T_1_	*p*‐value	T_0_	T_1_	*p*‐value	
Age (years)	67.2 [59.9; 72.9]			67.9 [60.3; 72.5]			0.92
Male, *n* (%)	22 (85)			22 (85)			0.99
Presence of COPD, *n* (%)	10 (39)			11 (42)			0.77
Lung function timeline							
Days from spirometry (T_0_) to surgery	18 [11; 33.7]			16 [6.25; 32.5]			0.47
Months from surgery to repeat spirometry (T_1_)	28.8 [15.7; 60.3]			40.8 [25.7; 56.7]			0.43
Surgical location							0.99
RUL, *n* (%)	2 (8)			2 (8)			
RLL, *n* (%)	7 (27)			7 (27)			
LUL, *n* (%)	9 (34)			9 (34)			
LLL, *n* (%)	8 (31)			8 (31)			
Surgery approach							
VATS/Open, *n* (%)	25 (96)/1 (4)			25 (96)/1 (4)			0.99
Number segments removed	1 [1; 3]						—
Mode of segmentectomy							
Complex/Simple, *n* (%)	11 (42)/15 (58)						—
FEV_1_, *L*	2.61 ± 0.79	2.44 ± 0.76	0.07	2.77 ± 0.66	2.25 ± 0.80	**< 0.001**	0.40
FEV_1_ (%) predicted	96.9 ± 30.2	92.6 ± 32.8	0.17	96.3 ± 17.8	79.3 ± 21.2	**< 0.001**	0.92
FVC, *L*	3.66 ± 0.82	3.34 ± 0.89	**< 0.001**	4.05 ± 0.81	3.43 ± 0.96	**< 0.001**	0.09
FVC (%) predicted	107.9 ± 25.2	101.7 ± 27.7	**0.042**	110.6 ± 18.6	94.5 ± 20.3	**< 0.001**	0.66
FEV_1_/FVC (%)	74.4 [65.8; 78.6]	72.1 [65.8; 77.3]	0.71	70.8 [61.7; 75.2]	65.9 [60.8; 71.5]	**0.01**	0.22
TLC, *L*	6.10 ± 1.19	5.84 ± 1.70	0.18	6.80 ± 1.34	6.02 ± 1.74	**0.004**	**0.046**
TLC (%) predicted	99.4 ± 16.8	94.7 ± 23.4	0.24	106.2 ± 17.2	92.9 ± 22.7	**0.003**	0.20
DL_CO_ (mL/min/mmHg)	19.8 ± 5.8	18.4 ± 6.5	0.18	19 ± 5.9	15.7 ± 6.1	**0.007**	0.65
DL_CO_ (%) predicted	80.5 ± 19.3	79.8 ± 17.8	0.87	73.6 ± 17.5	65.1 ± 21.9	**0.01**	0.19

*Note:* Data are shown as the number of subjects (in percentage), means ± SD, or medians (first quartile; third quartile). Significant *p*‐values are reported in bold.

Abbreviations: COPD, chronic obstructive pulmonary disease; DL_CO_, diffusion capacity for carbon monoxide; FEV_1_, forced expiratory volume at 1st second; FVC, forced vital capacity; LLL, left lower lobe; LUL, left upper lobe; RLL, right lower lobe; RUL, right upper lobe; TLC, total lung capacity; VATS, video‐assisted thoracoscopic surgery.

**TABLE 2:** Frequency of the segmentectomies procedures.

	Right (*N* = 9)	Left (*N* = 17)
S1‐2	0	1
S1‐2‐3	0	4
S2	3	0
S2, S4‐5	0	1
S3	0	1
S4‐5	0	2
S6	4	5
S6‐S8	1	0
S6b	0	1
S7‐8‐9	0	1
S7‐8‐9‐1	1	0
S8a	0	1

At T_1_, a non‐significant reduction in lung function measures was observed in the segmentectomy group, with the exception of FVC, which was modestly but significantly reduced. Conversely, in the lobectomy group, all lung function measures, including DL_CO_, were significantly impaired (Table 1).

## 4 Discussion

Preservation of lung parenchyma is attractive because it may be associated with reduced morbidity [5] and because lung cancer patients who were successfully operated on may develop a second primary lung cancer [3] that may be amenable to a second surgical resection. The relatively modest preservation of FEV_1_ after segmentectomy compared with lobectomy may be explained by distortion of the tracheobronchial tree in the spared lobe and by compensatory adaptations after lobectomy. Nonetheless, patients undergoing segmentectomy have been reported to experience less deterioration of self‐reported dyspnea scores over time [6] as well as faster recovery, better quality of life, and perceived dyspnea [5].

Four very recent reports [7, 8, 9, 10], also as updated meta‐analyses [8], evaluating the postoperative lung function after 3 [7], 6 [8], and 12 [9, 10] months document functional advantages of a surgical technique with more remarkable preservation of lung parenchyma. However, none of these studies assessed DL_CO_ [7, 8, 9, 10].

Few studies have reported changes in DL_CO_ after lung resection. In a multivariate analysis of 351 VATS lobectomies and wedge resections, Kim et al. [11] demonstrated that sublobar resection preserved DL_CO_ better 1 year after surgery. Similarly, Macke et al. [12] reported a correlation between the amount of spared lung parenchyma (i.e., 1–2 vs. 3–5 resected segments) and the preservation of FEV_1_ and DL_CO_. Chen et al. [13] found a significant difference in FEV_1_, FVC, and DL_CO_ preservation after segmentectomy vs. lobectomy, but only when less than 50% of the segments of the affected lobe were removed. In general, these data support the concept that segmentectomy preserves lung function better than lobectomy, provided that a minor portion of the affected lobe is removed. In our study, we similarly observed better preservation of nearly all lung function parameters including DL_CO_ in patients undergoing segmentectomy.

In the recent JCOG0802/WJOG4607L trial [2], a large prospective study comparing lobectomy and segmentectomy, with more than 500 patients in each arm, analyzed postoperative respiratory function changes at 6 months and 1 year. The median reduction in FEV_1_ was 10.4% at 6 months and 8.5% at 1 year for segmentectomy (*p* < 0.0001) vs. 13.1% at 6 months and 12% at 1 year for lobectomy (*p* < 0.0001) [2]. The differences in median FEV_1_ reduction between segmentectomy and lobectomy were 2.7% at 6 months and 3.5% at 1 year, although statistically significant (*p* < 0.0001), fell short of the predefined 10% threshold for clinical significance [2]. Although of clear scientific interest, this trial did not include DL_CO_ assessment in its lung function monitoring [2]. Nonetheless, with comparable lung cancer recurrence rate, overall survival was significantly better in the segmentectomy arm due to a reduction in competing causes of death [2]. In contrast, disease‐specific and overall survival were not different between lobectomy and sublobar resection in the CALGB 140503 trial [3], where a small, statistically significant difference was observed for FEV_1_ preservation in favor of the latter. Notably, lung cancer survival rates in the CALGB 140503 trial were much lower than in the JCOG0802/WJOG4607L trial, 65% vs. 85% at 5 years [2, 3].

Our study emphasizes the value of DL_CO_, a function of alveolar surface area, as a specific indicator of lung parenchyma preservation with segmentectomy. In a long‐term follow‐up (nearly 10 years), Ferguson et al. [14] showed that DL_CO_ is an independent significant determinant of survival. Tsubokawa et al. [9] further showed that a reduction rate in vital capacity—but not the FVC or FEV_1_—1 year after lobectomy or segmentectomy was an independent prognostic factor for overall survival (hazard ratio, 1.05; 95% confidence interval [CI], 1.02 to 1.07; *p* < 0.001). Therefore, postoperative function in terms of FEV_1_ probably does not reflect the full benefits of lung‐sparing surgery.

The major limitation of our study is the restricted sample size, reflecting the limited number of patients available for repeat lung function testing and matching. Moreover, only patients capable of performing repeat spirometry were included, which may introduce selection bias in favor of patients in good clinical condition; however, patients who repeated the spirometry served as their control within the same group. The strengths of our study are related to the exact matching of patients, particularly of the involved lobe, and the long interval before postoperative evaluation, after 35 months following lung resection, when compensatory adaptations have certainly become stable.

In conclusion, while we await further evidence, we propose that sparing the affected lobe by segmentectomy offers potential advantages in the long term—possibly due to better preservation of lung diffusion capacity, and segmentectomy, when feasible, should become the procedure of choice for early‐stage lung cancer.

## Author Contributions

Substantial contributions to the conception or design of the study and the acquisition, analysis, or interpretation of data: E.C., C.M., A.C., E.V., C.S., G.S., G.G., E.F., and M.I. Drafting the study or revising it critically for important intellectual content: E.C., C.M., and M.I. Final approval of the version to be published: E.C., C.M., and M.I. Agreement to be accountable for all aspects of the work in ensuring that questions related to the accuracy or integrity of any part of the work are appropriately investigated and resolved: E.C. and M.I.

## Ethics Statement

The study was conducted at the Thoracic Surgery Unit and the Respiratory Medicine Unit of the University and Hospital Trust of Verona (Italy). This study protocol was reviewed and approved by the local Ethics Committee (no. 3128CESC) and was carried out in accordance with Good Clinical Practice recommendations and the requirements of the Declaration of Helsinki. Written informed consent was obtained from all patients.

## Conflicts of Interest

The authors declare no conflicts of interest.

## Data Availability

The data supporting this study's findings are available on request from the corresponding author.
